# Misinterpreting carbon accumulation rates in records from near-surface peat

**DOI:** 10.1038/s41598-019-53879-8

**Published:** 2019-11-29

**Authors:** Dylan M. Young, Andy J. Baird, Dan J. Charman, Chris D. Evans, Angela V. Gallego-Sala, Peter J. Gill, Paul D. M. Hughes, Paul J. Morris, Graeme T. Swindles

**Affiliations:** 1 School of Geography, University of Leeds, Leeds, UK; 2 School of Natural and Environmental Sciences, Newcastle University, Newcastle upon Tyne, UK; 3 Geography Department, University of Exeter, Exeter, UK; 4 Centre for Ecology and Hydrology, Bangor, UK; 5 Geography and Environmental Science, University of Southampton, Southampton, UK; 6 Geography, School of Natural and Built Environment, Queen’s University Belfast, Belfast, UK; 7 Ottawa-Carleton Geoscience Centre and Department of Earth Sciences, Carleton University, Ottawa, Canada

**Keywords:** Ecological modelling, Hydrology, Wetlands ecology

## Abstract

Peatlands are globally important stores of carbon (C) that contain a record of how their rates of C accumulation have changed over time. Recently, near-surface peat has been used to assess the effect of current land use practices on C accumulation rates in peatlands. However, the notion that accumulation rates in recently formed peat can be compared to those from older, deeper, peat is mistaken – continued decomposition means that the majority of newly added material will not become part of the long-term C store. Palaeoecologists have known for some time that high apparent C accumulation rates in recently formed peat are an artefact and take steps to account for it. Here we show, using a model, how the artefact arises. We also demonstrate that increased C accumulation rates in near-surface peat cannot be used to infer that a peatland as a whole is accumulating more C – in fact the reverse can be true because deep peat can be modified by events hundreds of years after it was formed. Our findings highlight that care is needed when evaluating recent C addition to peatlands especially because these interpretations could be wrongly used to inform land use policy and decisions.

## Introduction

The peatland archive – obtained from the analysis of peat cores – has often been used to investigate the effect of changes in climate on long-term (centennial-millennial) rates of peat carbon (C) accumulation^1–3^. For example, compilations of peat core records are being used to examine the role of peatlands in the global C cycle, and to understand if they will shift from C sinks to sources in the future^4,5^. In the last few years, the most recent part of the archive, from near-surface peat, has also been used to interpret the consequences of different land uses on the C-sink function of peatlands (e.g.^6–8^). Because these interpretations are being used to aid decisions about land use by policy makers, there is a need to discuss the notion that highly dynamic near-surface peat can be reliably used for this purpose.

Peat has built up in peatlands over thousands of years^1^, providing an archive that has been used to i) reconstruct Holocene climate change^9,10^; ii) examine past landscapes and human impacts^11^; iii) identify important events such as the onset of the Anthropocene^12^; iv) elucidate climatic and ecological effects of volcanic eruptions^13^; v) understand how peatlands have developed through time^14^; and vi) investigate how land use affects C accumulation^6–8^. Studies using the peatland archive employ a range of proxies including peat humification, plant macrofossils, pollen and spore microfossils, geochemical indicators, and testate amoeba remains (e.g.^15,16^.). Age control is often provided by spheroidal carbonaceous particles (SCPs), tephra layers, short-lived radioisotopes (e.g. ^210^Pb and ^137^Cs) and radiocarbon (^14^C) to provide accurately-dated insights into past environmental and ecological change^17–20^.

### The challenge of interpreting C accumulation in peatlands

The rate at which C has accumulated in a peatland may be estimated by measuring the mass of C in the peat profile (from a peat core) and the age of the basal peat. If the mass per unit area of C is divided by the age of the peatland, the long-term rate of C accumulation is obtained (LORCA)^21,22^. LORCA will vary with the age of a peatland^21^ and cannot be used to indicate how a peatland’s C cycle has responded to past events such as changes in climate (e.g. the Medieval Warm Period and the Little Ice Age in NW Europe). For such finer-scale analysis, peatland scientists construct “C accumulation histories^3^”. Measurements of the age of peat at multiple depths down a peat core are used to construct an age-depth model for the peat profile. The C content of contiguous or regularly spaced samples of peat down a core is also measured. For each sample, or peat layer, a rate of C accumulation is calculated by dividing the C content (per unit area) of the sample by the difference in age between the bottom and top of the sample or layer. Using this approach, it is possible to see if changes in C accumulation through the core – through time – have occurred in response to external (allogenic) and internal (autogenic or developmental) factors.

Some studies^6,7^ have used fine-resolution estimates of recent C additions to the top of the peat profile (sometimes called RERCA: the recent rate of C accumulation^22^) as estimates of overall peatland C balance and have related these to recent land use change (see also Rydin and Jeglum^22^). In particular, both Heinemeyer *et al*.^6^ (p.7) and Marrs *et al*.^7^ (p.109) make inferences about changes in C accumulation rates over time, comparing very recently-formed peat to older material that accumulated decades to centuries earlier. However, palaeoecologists have known for some time that estimates of C accumulation rates in recently added peat cannot be assumed to be directly comparable to those derived from deeper peat.

**Figure 1:**
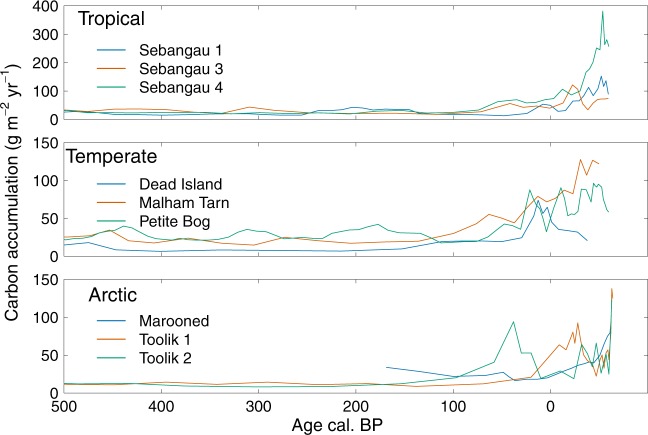
Carbon accumulation in three geographical zones. Data from peat cores showing recent apparent increases in the rates of C accumulation. 0 BP (before present) is 1950 CE (common era). Tropical peatland data are from Sebangau, Borneo^5^; temperate data are from Dead Island Bog, Northern Ireland, Malham Tarn, England and Petite Bog, Canada^5^; and the Arctic records are from Marooned, Sweden^30^ and Toolik, Alaska^29^ (see Supplementary Table S1).

Apparent increases in the rate of C accumulation are often evident in near-surface peat, but are an artefact^4,21,23–25^. The artefact arises because recently-formed peat has undergone less decomposition than older, deeper peat^21,23,24^. The mistaken assumption that rates of C accumulation in near-surface peat can be compared to those in deeper peat ignores the fact that much of the C in the uppermost peat profile will not become part of the long-term store^23,26^. Near-surface peat is in the zone in which the water table fluctuates (from the peat surface to depths of 50 cm or more) and in which oxygen is often readily available for decomposition: hydrologically and biochemically, the near surface is highly dynamic. Peat below this zone is subject to much lower rates of decay, mainly because there is less oxygen available (anaerobic rates of decay are very low in comparison to aerobic rates, often by a factor of 100 or more), but also because the remaining peat is more resistant to decay as the more readily-decomposed plant material has been lost^23^. As a result, contemporary C accumulation rates estimated for near-surface peat – the ‘acrotelm’^27^ – are many times greater than the long-term rates estimated for the deeper, saturated C store of ‘catotelm’^27^ peat (e.g.^21,23,26,28^). We show examples of this apparent recent increase in C accumulation rates for a selection of tropical, temperate, and Arctic peatlands in Fig. 1.

Whilst it is possible to attempt to correct for this artefact by applying constants for the rates of plant litter addition and decay^21,28^, palaeoecologists often deal with it by either ignoring the record from the upper part of a peat core^4,5^ or by combining estimated rates of C accumulation with other proxy data to qualitatively assess changes in the functioning of the peatland ecosystem^29,30^. A second important consideration is that deeper peat can be modified by events such as drought or fire that occur many years after it was formed^26,31^. For obvious reasons, short cores cannot reveal what is happening, or what has happened, in such deeper peat. Therefore, although short cores can be used to compare spatial differences in recent rates of C addition over a few decades (e.g.^7,24^), they cannot be used to determine if a peatland is currently a C sink or a source because they contain no information about C losses deeper in the peat profile. Incomplete decay of newly added plant litter and the continued modification of deeper C stores means that contemporary C addition to the acrotelm should not be used as a measure of temporal variability in long-term C accumulation rates. As a result, understanding the effect of climate change on recent rates of C accumulation in peatlands, based on data from cores, is one of the current challenges in palaeoecology.

### Simulating peatland C accumulation

Here we use the DigiBog peatland development model^32,33^ to show in more detail how the near-surface artefact in the apparent rate of C accumulation arises. We explore how using the upper profile alone for estimating a peatland’s C budget can give a fundamentally flawed impression of the impact of management or climate on peatland functioning. In particular, we simulate the effect of ditch drainage to show how C additions to the upper part of the peatland can occur despite an overall loss of C from the peat profile. We ran two simulations; one of an intact (‘natural’) peatland and one where a series of regularly spaced ditch drains was added (see Methods) along the transect between the centre and the margin of our peatland (‘drained’).

**Figure 2:**
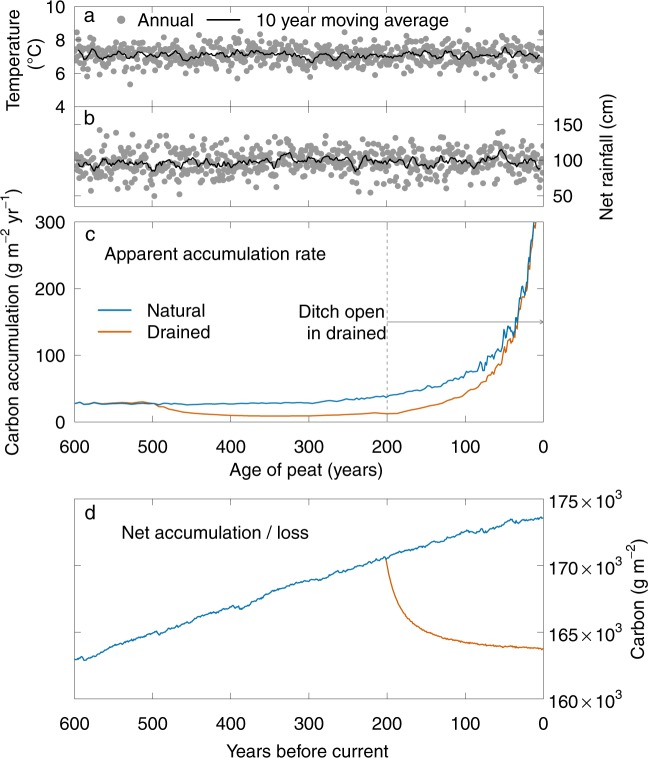
Simulated rates of C accumulation in the natural and drained peatlands. Driving data: (**a**) annual air temperature, and (**b**) the time series used to create the net rainfall input. (**c**) Apparent C accumulation rates and (**d**) net C accumulation/loss. The peatland was modelled over 6,000 years (most recent 600 years shown). For the ditch-drained simulation, a series of 0.5 m deep ditches were added 200 years before the end of the simulation (before a notional ‘present’) and remained open (i.e., they were open for 200 years). See Methods for details of the model parameters and the driving data.

In DigiBog, a peatland consists of contiguous and hydrologically connected columns of peat. These columns grow upwards if the rate of plant litter addition exceeds the rate of peat decay in the column. On the other hand, if decay exceeds plant litter addition, columns shrink and the simulated peatland surface subsides. Rates of litter addition depend on water-table position, which is simulated using a hydrological sub-model within DigiBog, and on air temperature (Fig. 2a). Water table and temperature also affect rates of peat decay, which are typically much higher above the water table than below it (see above). Water-table position depends on climate (net rainfall – Fig. 2b) and the hydraulic conductivity (permeability) of the peat which is also simulated by the model (see Young *et al*.^33^ for a full description).

The C accumulation history (see above) in a ‘virtual’ core taken from the midpoint between the centre and margin of our modelled natural peatland (Fig. 2c) is comparable to the peatland core data shown in Fig. 1, and the long-term rate is within the range of values published elsewhere (e.g.^5,24,26,34^). For example, Gallego-Sala *et al*.^5^ reported mean C accumulation rates from CE 850–1850 of 3–80 g m^−2^ yr^−1^ for a range of peatlands: the rate for our virtual natural core for a similar 1,000-year period was 26.9 g m^−2^ yr^−1^. The modelled ‘natural’ peatland shows that, although long-term C accumulation was relatively constant (Fig. 2c), the apparent rate of C accumulation in near-surface (recently added) peat was significantly higher than in peat that was more than 200 years old (i.e. the long-term rate) (Table 1).

**Table 1:** Apparent rates of C accumulation in cores from the centre-margin midpoint of the natural and drained peatlands. For details of the simulations, see Methods.

Age of peat (yrs)	Natural (mean rate g C m^−2^ yr^−1^)	Drained (mean rate g C m^−2^ yr^−1^)
0−200	139.7	119.8
200−600	29.6	15.4

The results of the ditch-drained simulation shows how deepening the water table leads to the net loss of C from the peatland, shown by the line of C accumulation for the drained core being below that of the natural core (Fig. 2c,d) (see, also, Table 1). Deeper peat that has accumulated several centuries before the peatland is drained loses mass only very slowly under anoxic decay conditions. However, when the ditch is added, and water tables are deepened by drainage, this peat undergoes relatively high rates of decomposition because it is exposed to oxic conditions once again (known as secondary decomposition^31^). This is why the drained line in Fig. 2c,d is below the natural line.

Our results also show that, whilst these deeper (older) C stores are being depleted, new C can still be added to the peatland surface at rates that appear to have increased when compared to the long-term rate of accumulation (Table 1). Table 1 shows that the rates of C accumulation calculated for peat less than 200 years old are many times higher than that calculated for older peat. However, peat that is less than 200 years old is relatively close to the surface of our simulated peatland – its thickness in the natural and drained cores is 0.42 m and 0.33 m respectively – which is still in the zone of water-table fluctuation and it could therefore be periodically subject to the higher rate of oxic decay. The net result of these processes (the balance between plant litter addition and peat decomposition) under our simulated ditch drainage conditions, is that the total amount of peat (and therefore the carbon stock – see Fig. 2d) decreases even though new litter/peat continues to be added to the peatland surface. At the end of the simulations, the thickness of the peat core from the natural peatland was 3.47 m, whereas the peat core from the drained peatland was 3.28 m. Frolking *et al*.^35^ used a 1D model to compare apparent C accumulation rates and peatland C budget in response to different precipitation inputs and a ditch drain (simulated by lowering the water level within the peat column). Similarly, they also showed a discrepancy between the two quantities and highlighted the challenges of making inferences about net C balance from the apparent accumulation rate. Both modelling approaches show clearly why it is a mistake to use recent rates of C addition to the upper part of a peat profile as an indication of overall peatland C accumulation rates, or of net peat C balance^6,7^.

### Comparing contemporary and long-term rates of C accumulation

Whilst it might seem obvious that ditch drainage causes a peatland to lose mass overall (e.g.^36,37^), our simulated peatlands illustrate two important points about how observations based on the rate of C accumulation in near-surface peat can be misleading.

Firstly, the partially decomposed plant litter that is added to a peatland does not remain a fixed quantity. As has been known for some time^27^, plant litter added at the surface decays at an initially rapid rate, which slows as the residual material becomes less degradable, and is gradually buried within the peat profile. Eventually it becomes waterlogged where decay of the remaining, largely recalcitrant, fraction continues very slowly. By this point, only a small amount of the original material remains as part of the long-term C store: our simulations reproduce this effect (see also Frolking *et al*.^35^). It therefore follows that, even in the absence of any changes in climate or peatland management, the calculated rate of C accumulation in near-surface peat will be many times higher than that in centuries-old material. This roughly ‘hockey stick’ shaped profile is what palaeoecologists would expect to see in their C accumulation profiles (Fig. 1).

Our simulations demonstrate that long term and recent rates of C accumulation cannot be compared directly in a useful way without considering the incomplete decomposition of the near-surface peat. Attempts to take account of the artefact include Loisel and Yu^28^, who used three models parameterised with data from their cores (from a site in south central Alaska undergoing a fen-to-bog transition) to calculate decomposition losses in acrotelm peat which they compared to older catotelm peat that they ‘recomposed’ using reconstructed fluxes. Whilst this approach is a significant improvement on direct comparisons of recent and past estimates of rates of C accumulation, additional data from cores is still needed to contextualise model outputs and test their plausibility^28^. However, if meaningful comparisons are to be made between recent and long-term estimates of C accumulation rates, an ecological modelling approach is probably needed.

**Figure 3:**
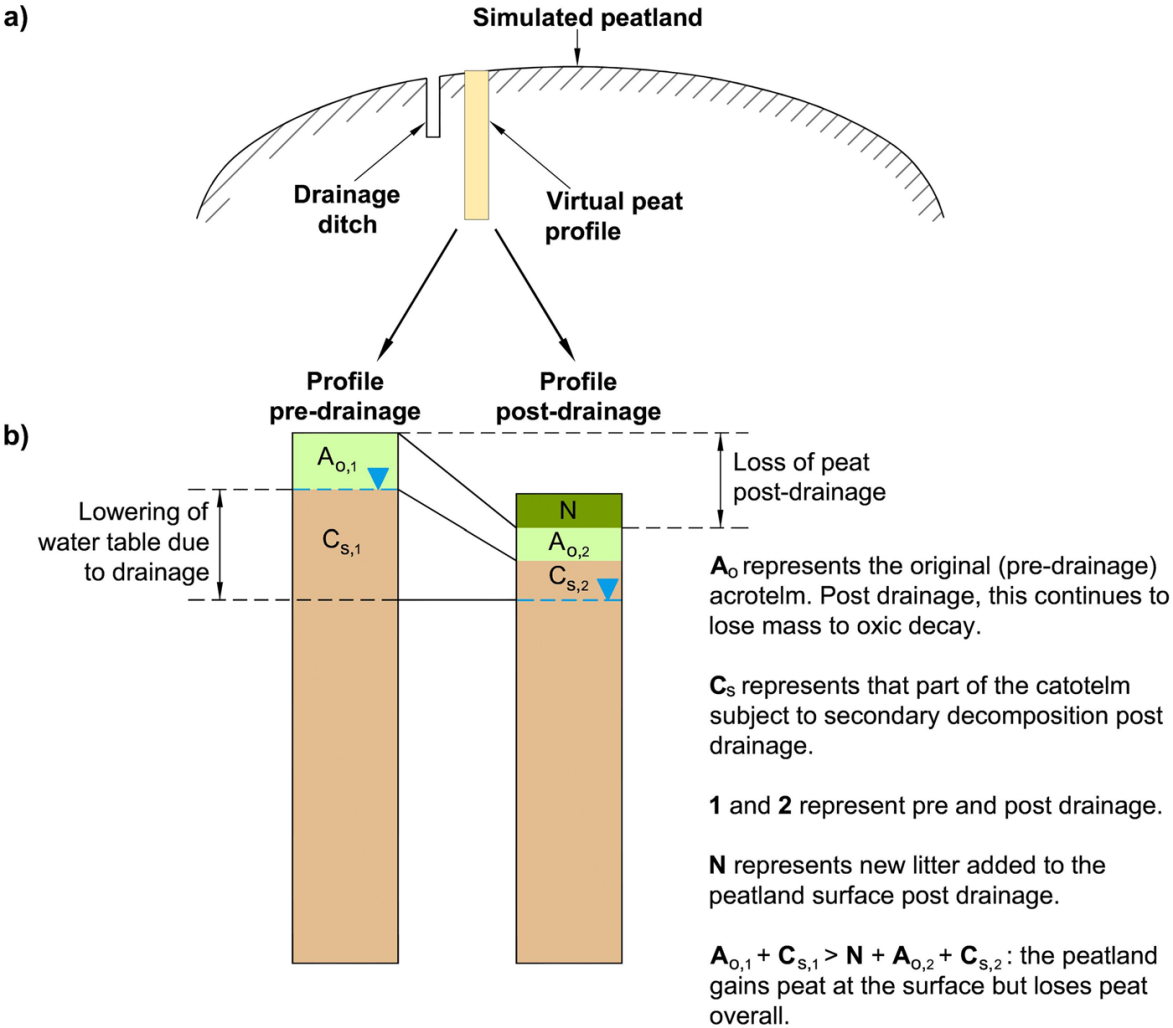
The change in the C store of a simulated peatland before and after ditch drainage. (**a**) Cross section of peatland showing ditch drain and nearby virtual peat core. (**b**) The reduction in C store caused by a ditch drain. Although new peat is added to the surface of the example column, the total loss of peat throughout the column due to decay in both the original acrotelm (A_o_) and the original upper catotelm (C_s_) exceeds the mass of the new material (N).

Secondly, our ditch-drained simulation demonstrates that high rates of litter addition at the peatland surface do not indicate that the peatland as a whole is accumulating C – the addition of new mass needs to *exceed all losses throughout the whole profile* for this to be the case^23,24^. This point is explained further in Fig. 3. Therefore, near-surface rates of C addition cannot be used on their own to represent the overall C budget of a peatland^24,26,28^ and should not be directly compared to the rates of accumulation from other peatlands where the total C budget has been correctly measured^7^. This is especially the case where management or random events such as drought or fire^26^ can alter the decay rate of deeper peat by exposing previously anoxic material to aerobic (secondary) decomposition, as shown in our ditch-drained simulation.

Our results also have relevance to the ongoing debate regarding the impact of drainage-based plantation forestry on the peatland carbon balance. Studies in Fennoscandia and in the UK (e.g.^38–43^) have suggested that peatland forestry may be net-beneficial for C stocks due to the higher rates of litter input compared to a natural bog. Our simulations suggest that, by quantifying the (easy to measure) accumulation of near-surface litter but not the (hard to measure) decrease in deep peat C stocks, these studies may have reached overly optimistic conclusions regarding the net impact of forestry activities on the peatland C balance.

Clearly, policy makers require timely information about the impacts of climate and land use on peatland C stores, but our model simulations demonstrate that assessments based on near-surface peat alone are likely to be misleading at best. In contrast, measurements of CO_2_ exchange across the peat surface (e.g. using eddy covariance techniques) capture the net effect of changes in both the input of C to the system and in the overall rate of decomposition throughout the peat profile. While these methods also present methodological challenges (notably requiring measurements or estimates of fluvial dissolved and particulate C losses to construct whole-peatland C budgets), they are likely to provide the most reliable indication of the net impact of management activities on the contemporary peatland C balance, especially when averaged over a sufficient number of years to account for inter-annual variability (e.g.^44^).

Care is also required when comparing contemporary C flux measurements to data from deeper within peat cores. Even when near-surface peat is ignored, C accumulation rates calculated for discrete layers do not indicate the whole-peatland C budget for the time period represented by the difference in age between the bottom and top of the layer. For example, consider a peatland that formed 9,000 cal. BP and a layer of peat within that peatland that is 4,300 cal. BP at its base and 4,180 cal. BP at its top. The calculation of the C accumulation rate for the layer simply considers organic matter added to the peatland between the bounding dates and what has happened to that organic matter over the years since. However, between 4,300 cal. BP and 4,180 cal. BP, C would have been lost via decay from the peat that formed between 9,000 cal. BP and 4,300 cal. BP – the older peat below the layer – and this loss would have been part of the whole-peatland C budget at the time. Necessarily, when we consider only the organic matter within a particular layer, the C budget of the rest of the peat profile is ignored. Therefore, C accumulation rates calculated for layers of peat cannot be meaningfully compared with contemporary flux measurements.

It may seem reasonable instead to compare contemporary flux measurements with the average rate of peat accumulation estimated for the entire peat profile (LORCA), because both consider the whole peat column. However, LORCA averages across a peatland’s entire developmental history (likely to be over many centuries), whereas gas flux measurements are quasi-instantaneous (one or two decades at best). This difference in averaging periods leads to two problems that prevent useful comparisons. Firstly, LORCA is affected by past changes in external boundary conditions (e.g. climate, drainage), whereas flux measurements only reflect how modern external conditions are influencing a peatland’s C budget. Secondly, LORCA contains information – for example, peat thickness, C stock, vegetation type, and trophic status – generated when the peatland itself was almost certainly different from today (see^21^). However, in contrast, contemporary gas fluxes reflect only the peatland’s current status. For these reasons, LORCA and contemporary gas flux measurements describe peatlands that may be very different from one another. As such these two measurements cannot be compared in a meaningful way.

## Conclusions

Our findings show that the growing use of the uppermost part of the peatland archive for environmental reconstruction should be tempered with caution on what this part of the peat profile can reveal about the C-sink function of peatlands. Our results support the recommendation that C accumulation rates obtained from peat cores should not be used as the only source of evidence about the recent effects of management or climate on the peatland C store, but should be coupled with other models and contemporary and/or palaeoecological evidence about the ecosystem (e.g.^26,28,29^). Our numerical experiments and discussion highlight:

1. Rates of net C addition in near-surface peat cannot be directly compared with long-term rates from deeper layers.
2. A peatland can undergo net C loss even though the uppermost part of the profile shows a gain in C.
3. Spatial comparisons of recent C addition can be made using short cores but cannot be used to infer the total peatland C budget.
4. Contemporary C flux measurements cannot be directly compared to C accumulation rate estimates for peat layers from the palaeoecological record.
5. The average rate of C accumulation over the entire developmental history of the peatland (i.e. from the base to the surface, LORCA) cannot be meaningfully compared with contemporary flux measurements due to the substantial differences encompassed by their averaging periods.

## Methods

### Model setup and accumulation rates

We used DigiBog to simulate the accumulation of peat over 6,000 years in a small 300-m wide raised bog on a flat impermeable mineral soil – we simulated a 150 m transect (75 × 2 m × 2 m columns) from the centre of the peatland to its margin where a fixed water level represented a lagg stream. In the ditch-drained simulation, ten 0.5 m deep contour-parallel ditches were simulated for the last 200 years of the model run. Starting at 10 m from the peatland margin, the ditches were simulated at 12 m intervals. Ditches were created in the model by removing the peat layers in each of the ditched columns and setting a Dirichlet constant water level^33^ at 0.5 m below the peat surface. The model was set up as described by Young *et al*.^33^ but with the parameter values from Morris *et al*.^45^ (who simulated the raised bog of Malham Tarn Moss in northern England – henceforth MTM – using a 1D version of DigiBog). We used the version of DigiBog described by Young *et al*.^33^ (available with a set of input files from https://github.com/youngdm/Digibog_PDM_WRR2017) with the addition of a routine to speed up run times by aggregating layers of peat of less than 2.5 × 10^–3^ m thick.

In DigiBog, new peat layers are added to each column at the end of every year of a simulation. Within a year layers are decomposed according to water table position and annual air temperature. Throughout a simulation, the model keeps track of the age and the decreasing mass (g m^−2^) of a layer which enabled us to calculate the annual rate of peat accumulation between two contiguous layers. Starting at the base of a virtual core, we added half of the mass of sequential pairs of layers (i.e. layers 1 and 2, layers 2 and 3, etc.) and divided the sum by the number of years between the two layers. To estimate the rate of C accumulation, the resulting peat mass was multiplied by 0.5^28^.

### Driving data

The climate inputs to the model (rainfall minus evapotranspiration and annual air temperature) represented the Holocene climate around MTM (see Morris *et al*.^45^ for a description of the location and reconstruction of the climate inputs). We used 6,000 years of the reconstructed data: the 6,000 year mean net rainfall and air temperature was 96.2 cm and 7.1 °C respectively (Fig. 2a,b). Although the original net rainfall data series was reconstructed as annual inputs, rather than use this lumped value we imposed a seasonal pattern on the data and distributed them as weekly inputs to DigiBog. This approach allows excess winter net rainfall to be lost from the model (analogous to runoff) rather than to be effectively redistributed to drier summer months.

We modified the MTM annual net rainfall data with weekly inputs from data collected between 2010 and 2013 at Keighley Moor (henceforth KM) – also in northern England – used in DigiBog simulations by Young *et al*.^33^. The KM data represented a single average year; therefore, although the annual net rainfall for MTM varied from year-to-year, we imposed the same seasonal *pattern* from the KM data across all MTM years. Each annual value from the MTM time series was transformed into a weekly series by first processing the KM data. We calculated the proportional difference between the KM weekly values and the KM annual mean for each of the 52 weeks in the dataset. Then, for each year of the MTM dataset, we multiplied the annual net rainfall by each of the 52 KM proportional differences to produce weekly values for 6,000 years.

## Supplementary Information

Supplementary Information

## Data Availability

DigiBog model outputs are available from Dylan M. Young on reasonable request. Peat core C accumulation data is in the Supplementary Information.
