# Supplementary material for: Misinterpreting carbon accumulation rates in records from near-surface peat
Source: Sci Rep. 2019 Nov 29;9:17939. doi: 10.1038/s41598-019-53879-8 (PMC6884541; doi:10.1038/s41598-019-53879-8)
Supplement: Supplementary file 1 — Supplementary Information [file 41598_2019_53879_MOESM1_ESM.pdf]

Misinterpreting carbon accumulation rates in records from near-surface peat

Dylan M. Young, Andy J. Baird, Dan J. Charman, Chris D. Evans, Angela V. Gallego-Sala, Peter J. Gill, Paul D. M. Hughes, Paul J. Morris, Graeme T. Swindles

Supplementary Table S1. Example carbon accumulation records for tropical, temperate, and Arctic peatlands

(bp = before present, C = carbon accumulation rate)

Tropical						Temperate						Arctic					
Sebangau1_bp	Sebangau1_C	Sebangau3_bp	Sebangau3_C	Sebangau4_bp	Sebangau4_C	Dead_Island_bp	Dead_Island_C	Malham _Tarn_bp	Malham_Tarn_C	Petite_Bog_bp	Petite_bog_C	Marooned_bp	Marooned_C	Toolik_TFS1_bp	Toolik_1_C	Toolik_2_bp	Toolik_2_C
-59.4	89.33933922	-59.2	73.88792423	-59.9	257.2075973	-37.7	20.74579287	-49.6	122.1116192	-59.6	58.45588004	-62	92.1726	-64	125.3333349	-63	124
-57	136.2461042	-55.6	71.83548189	-57.8	280.6982205	-27.8	31.84372184	-43.3	126.6823262	-57.8	61.55820759	-61.12	79.4115	-63	137.8	-60	24.89130484
-54.6	116.1295507	-52	71.83548189	-55.7	263.0989849	-18	33.76498108	-37.1	107.0598177	-56.1	68.48759049	-58.6	75.0794	-62	78.66666667	-57	51.84210484
-52.2	152.4283915	-48.3	69.89398238	-53.7	381.4017078	-8.3	35.38577339	-30.8	127.6484943	-54.3	75.13147003	-55.03	64.6518	-60	44.125	-52	26.15
-48.7	107.6872387	-43.8	57.46838551	-50.8	245.0823944	-1.2	44.94489243	-23.1	82.50920016	-52.6	91.09704113	-50.7	49.8002	-59	56.90909304	-47	65.88235311
-43.9	83.61025649	-38.4	33.92400585	-47.3	251.6850709	3.3	64.72465574	-14	87.26158285	-50.9	93.17520892	-44.52	39.8824	-57	52.85714186	-43	34.54861151
-39.1	112.8751615	-33.1	54.64468377	-43.7	202.0469435	7.9	56.36773224	-5.1	76.48301159	-49.2	95.10274082	-39.59	40.9322	-55	32.73684149	-38	51.84126961
-34.2	83.40488578	-27.9	106.5372921	-40.1	177.6112807	12.7	73.90196983	3.9	71.76094151	-47.5	91.33545386	-35.03	38.8538	-53	50.27173893	-32	64.20740741
-29.4	65.31895805	-22.6	121.3765198	-36.5	166.0964043	17.2	52.99368464	13	79.04827778	-45.9	93.00317992	-28.01	36.4525	-51	44.1666659	-24	18.9453125
-23.1	64.86134692	-12.9	57.78190901	-30.4	100.282868	29.6	24.37076735	23.9	64.46025507	-44.2	96.34569168	-21.48	32.6631	-47	22.26923033	-10	29.06799076
-15.4	31.19486565	0.7	39.57522486	-21.4	86.24248369	50.1	19.29526538	37.2	44.05381871	-41.7	71.60769481	-14.21	29.2173	-43	38.45899451	10	19.26742531
-7.7	28.47963675	14.3	47.16199305	-12.3	106.453542	70.5	20.11124661	50.2	50.30105911	-39.2	79.93814456	-6.38	24.9898	-38	42.63313584	20	52.9143491
0	49.56546078	28	42.55137819	-3.3	73.44311712	90.8	20.06622308	63.3	55.2320943	-36.7	87.73545287	3.3	19.9893	-34	50.39251204	30	52.73504271
7.8	53.59293729	41.6	56.04607146	5.7	69.42547484	111	19.46789566	76.3	42.86175385	-34.2	88.15404371	13.8	18.1168	-31	69.40144517	38	94.10244366
24.2	20.99419364	71.6	26.87290151	15.1	59.92652368	152.6	9.644143415	99.7	30.04719433	-31.7	88.56975714	25	18.112	-28	92.54709238	58	40.66425125
47.8	12.95300405	116.9	17.79799729	24.9	58.17786854	215.2	6.778819149	135.7	19.80152145	-28.2	59.19477238	36.44	16.3532	-25	65.61032824	97	20.19230767
71.1	16.04754443	161.2	21.9096389	34.7	69.41547129	277.2	7.53296869	170.5	18.84397651	-24.7	55.12691245	42.83	21.5371	-23	80.35294158	154	12.20969091
94.6	17.74637769	205	20.81449301	44.5	66.15797057	339.1	8.263088647	205.2	17.09125576	-21.3	55.83198232	48.63	27.391	-20	70.23809481	228	8.514668514
118	17.72071204	249.2	23.56761006	54.3	62.30429763	401.1	6.421130927	240	20.59117636	-17.8	53.32921602	66.84	23.1905	-16	57.04671979	302	8.10060059
135	21.39703909	283.8	32.01122793	73.8	33.1120611	446.6	8.406541978	273.3	24.96387199	-14.4	76.15496446	100	21.8991	-9	63.60229297	376	9.242627718
146	35.3772017	309.5	43.4330518	101.9	24.18005114	474.8	17.96529167	304.8	14.68909266	-10.7	90.44704717	134.64	28.4194	20	20.82254331	432	12.6358696
157.4	35.16439968	338	21.29932783	128	22.57052372	502.2	14.5297695	337.9	17.13804979	-7	76.78673917	168.54	33.8355	69	12.14053802	486	12.08380096
169.8	36.32051557	366.4	25.49748365	153.3	31.74448792	530.2	15.29200388	372.5	23.86661862	-3.3	63.61809212			138	8.767383962	539	14.4549266
182.2	33.13899511	394.1	34.23509084	178.8	29.02243116	558.2	13.3605476	407.5	17.4939103	0.4	48.40369526			188	12.43454546	591	12.02152016
193.1	40.31396359	421	36.62587586	206.5	19.26628489	583.4	16.30715658	433.7	20.4309727	4.2	32.23752724			239	11.04236263	643	10.81382382
203.7	42.63256048	448.2	36.33899002	237.1	23.16104231	605.6	18.77684422	453.2	34.58092007	7.6	49.39236324			291	14.36174424	695	12.70702982
214.6	34.0953361	475.6	27.9345247	268.5	20.5919587	627.9	19.32352038	473.3	27.04534312	11	62.7076976			343	11.43253705	747	13.67294057
226.7	31.05792928	501.6	33.77121134	299	23.8435475	650.2	17.22816373	494.3	25.47469778	14.5	67.57636187			395	14.35746606	798	13.44019089
238.9	30.82162534	522.6	46.19643025	328.4	19.61129755	672.9	18.21410773	515.7	24.89484078	17.9	76.65627943			449	10.86369385	850	10.77008931
256	15.01602942	547.1	29.4764256	354.6	24.83755957	699.4	13.1471637	548.8	12.22463455	21.3	87.6217118			502	11.55511421	904	13.46869967
276.5	14.97184678	576.2	26.92078827	378.3	24.7857855	729.2	9.881356324	595.8	13.21152091	30.7	35.57982881			556	11.58888889	960	14.52876982
293.1	17.00607908	609.5	26.65195601	403.7	24.25733006	759.2	12.00100806	644.3	10.89875084	39.2	40.42794333			610	12.03409758	1019	12.60724001
310.5	19.94018591	646.5	27.11606413	430.1	22.90468694	788.2	12.96210859	693.4	9.620183247	47.5	42.49794549			662	15.41025643	1076	15.95337027
328.2	20.08876884	677.9	32.23681423	454.9	23.98360063	817	11.87106813	741.1	9.488294094	55.8	34.24062609			715	13.37386017	1133	12.75738366
348.3	19.18696303	725.1	32.8734995	476.3	23.59672021	846.5	9.874236628	774.1	16.85170825	64.3	25.45685153			767	14.49091029	1191	12.51231527
373.1	16.69159328	795.8	16.16556208	496.6	31.58647815	875.8	10.9481546	794.2	30.04371724	76.1	19.36881775			817	14.065	1250	13.23637199
398.7	14.98943866	868.5	23.42460289	517.3	27.04860417	905	14.19922209	816.1	27.65484989	88.6	19.26352486			859	19.95226994	1308	14.30818966
423.1	16.81031291	941.7	22.47130087	537.3	26.88868573	934.1	12.31780889	838.9	24.00458707	100.8	18.37006181			893	21.36222906	1366	12.42380706
447.5	17.41603108	1015.4	20.32370358	556.5	31.26750238	963.6	9.010921865	861.8	25.3072405	112.6	17.58100636			925	26.09479521	1423	13.28480183
468.9	25.01568097	1092.6	18.88139976	575.4	34.02651475	991.3	10.93024256	878.6	31.04482865	123.3	24.50666327			957	25.42858544	1480	13.2052353
489.8	27.63179635	1174.5	21.13977791	594.8	33.17898001	1014	11.86099514	890.1	52.40979019	133.8	30.23584716			989	27.45238092	1536	10.55916308
512.6	23.37650522	1259.8	15.52953865	614.8	32.95456319	1037.3	13.26958445	902.3	39.22509424	144.6	30.59071356			1021	26.5375	1586	15.51820731
535.9	26.06596717	1348.7	14.95799796	635.1	26.80407368	1059.7	12.26344501	914.3	52.59903607	155.7	30.94819267			1052	34.52760421	1636	17.09593023
559.3	19.47218612	1435.3	13.71897153	655.4	26.83397149	1082.9	11.85505244	925.9	40.04157973	167	34.3029503			1084	27.28847107	1686	17.84191177
579.5	24.33154493	1490.3	22.80515297	673.3	28.63298924	1108.7	11.31340127	936.5	43.97928111	177.2	42.05117545			1109	39.56085112	1736	17.45721928
598	26.38993255	1528.7	26.30699475	689.9	28.93414229			946.4	50.69468499	187.2	39.07344898					1786	17.90505048
619.1	18.67920447	1567.2	31.02350237	706.4	23.04193166			956.1	53.93643342	197.2	35.06379727					1836	35.2631579
645.8	18.15755981	1595.2	41.75421208	722.9	31.00946152			965.9	61.02119166	206.6	34.66646611					1886	16.86545454
668.3	21.49901727	1619.7	44.04620441	741.5	31.49266888			975.8	46.4240816	215.9	32.45006008					1937	18.80392156
688.3	25.27029687	1641.1	58.21364458	762.1	28.73400291			985.9	42.67097337	224.6	30.2290617					1987	15.66031744
709.7	25.3603667	1660.9	56.42264994	783.9	24.00325159			997.1	47.37016711	234.2	23.49246468						
731.3	21.85862185	1679.1	61.16722563	803.5	37.09109855			1008.9	41.91192244	244.4	23.57377028						
751	22.22667163	1696.1	60.20618641	822.8	34.71703223			1020.4	37.27079395	254.7	24.81483688						
771.1	25.21049319	1711.6	87.7755897	841	44.453565			1031.9	48.04160293	264.5	23.93487483						
790.1	22.80824176	1729.6	49.33555298	860.9	36.60295551			1042.9	61.25077195	273.8	22.98417408						
810.3	24.68372067	1750.2	64.24340712	883.7	35.03131282			1054.2	58.60237894	283	27.7355302						
830.5	24.44236684	1770.8	63.71238659	905.9	34.57995772			1065.2	50.48395603	292.2	32.31382148						
850.2	25.06679983	1790	50.80879175	927.4	35.77991542			1076.1	50.918931	302	33.06849974						

Misinterpreting carbon accumulation rates in records from near-surface peat

Dylan M. Young, Andy J. Baird, Dan J. Charman, Chris D. Evans, Angela V. Gallego-Sala, Peter J. Gill, Paul D. M. Hughes, Paul J. Morris, Graeme T. Swindles

Supplementary Table S1. Example carbon accumulation records for tropical, temperate, and Arctic peatlands

(bp = before present, C = carbon accumulation rate)

Tropical						Temperate						Arctic					
Sebangau1_bp	Sebangau1_C	Sebangau3_bp	Sebangau3_C	Sebangau4_bp	Sebangau4_C	Dead_Island_bp	Dead_Island_C	Malham _Tarn_bp	Malham_Tarn_C	Petite_Bog_bp	Petite_bog_C	Marooned_bp	Marooned_C	Toolik_TFS1_bp	Toolik_1_C	Toolik_2_bp	Toolik_2_C
869.4	30.19788591	1809.4	45.97393506	948	41.61712064			1086.1	58.66776784	311.9	35.41647438						
890	26.26452896	1827.9	28.60865146	968.5	41.68366151			1099.9	59.93833378	321.3	31.67418171						
910.9	28.87339334	1849.3	29.29664686	990.4	37.42291553			1117.6	32.69407183	331	25.15765609						
931.6	33.16849708	1868.9	39.32046971	1013.2	40.19624484			1134.6	39.23500061	341.6	22.20556143						
951.7	34.1872938	1889.4	58.12850519	1036.8	42.72812643			1151.7	39.83534677	352.3	21.19569193						
973.2	29.06694532	1909.1	47.45751303	1060.7	36.91081572			1168	38.635233	362.8	21.17460251						
1124.9	4.595431624	1928.3	40.22817732	1101.3	27.36286797			1190.7	25.30918464	372.8	21.79156147						
1366.8	3.453042673	1949.5	35.05265116	1156.1	19.14371826			1219.8	18.51130077	381	23.66519144						
1598.3	3.120083846	1969.9	42.39816632	1208.5	19.30561914			1247.1	17.52558081	389	21.27422901						
1828.3	2.949118566	1989.3	25.56073181	1260.2	20.72761383			1276.7	17.97756502	397.2	22.11463306						
2055.2	2.78434987	2008.2	44.19569688	1309.4	18.37719096			1303.7	17.24878106	405.5	23.19683682						
2340.9	3.450505799	2027.1	40.51278879	1366.1	15.43696707			1337.1	14.54204679	414	26.52028328						
2699.8	2.001666969	2047.5	26.556473	1435.9	14.49106832			1381.6	12.99779669	422.1	31.87286367						
3086.8	1.702933706	2068	32.73525389	1506.9	13.39862243			1426.7	10.42041083	430.1	37.39586487						
3457.5	1.694397021	2086.6	33.48651892	1572.9	17.54912587			1474.1	8.545719623	438.7	39.76837994						
3779.2	2.182444498	2105.1	31.74914658	1635.8	16.27463626			1518.7	10.54837772	447.6	34.2800724						
4078.2	2.228863508	2123.5	29.15437153	1705.9	15.34214718			1559.8	11.14830091	455.8	32.83251659						
4373	1.992433285	2143.1	34.9024338					1597.7	12.33210905	463.6	30.87774346						
4663.8	1.058343294	2163.1	27.5020337					1639.1	14.24389941	471.8	25.93960767						
4957.7	1.314774669	2182.8	34.55798771					1682.6	10.48345931	480.7	23.73992687						
5281.2	1.350531616	2200.7	31.28160126					1721.6	12.77684429	489.7	23.31896296						
5486.1	3.218834783	2220.2	29.66283826					1756.1	16.8285338	498.5	21.55064297						
5564.2	13.26706019	2242.4	26.22343968					1789.1	11.68676077	505.4	24.58906503						
5657.2	9.962681275	2264.1	14.50262791					1822.9	11.69103725	512.1	32.20228604						
5728.6	11.66015576	2284	13.07870694					1856.6	14.26332801	519.1	37.37324839						
5795.1	12.3324769	2305.2	13.15301321					1888.1	17.39604639	527.2	29.66810852						
5849.8	10.79751571	2323.8	14.26045816					1917.4	15.32555825	534.9	28.40954059						
5901.8	10.27418477	2344.1	19.91226431					1946.2	17.00589085	540.2	45.81716723						
5947.3	16.04511797	2363.2	26.85268582					1975.2	17.42203138	546	46.1839821						
5989	17.37992302	2381.8	19.33741274					2006.5	18.93874342	551.8	48.00592638						
6029.8	16.3701963	2400.7	30.56638822					2037.7	12.17777685	557.6	49.73140387						
6078.6	14.79504425	2420.3	21.58522466					2062.3	17.84154209	562.8	46.88678858						
6129.1	13.01689503	2440.7	31.69598741					2084	19.9637292	567.7	40.40068129						
6178.1	11.57965326	2461.4	16.07716606					2105.4	13.6394275	572.7	49.48040395						
6224.5	15.67805647	2480.6	25.92638838					2126.3	20.71102778	577.5	61.80464072						
6272.5	13.57237572	2501.8	28.84374595					2146.5	19.59662171	582.2	58.21264012						
6322.4	11.4451849	2521.3	31.50275659					2166.9	11.56417418	586.7	55.80509649						
6372.3	11.26424734	2541.1	25.15994251					2188.3	13.18059799	591.8	49.03131817						
6421.6	10.17055555	2560.2	23.2899247					2209.2	11.7121575	596.7	50.80589275						
6472.7	6.312554541	2581.2	20.14470954					2228	27.82549166	601.8	58.1966922						
6522.5	7.527281325	2601	26.37876472					2245.5	29.38855439	606.5	73.28711976						
6571	10.67243172	2620.8	22.58671284					2262.8	38.05780346	611.2	68.38907812						
6621.5	6.602685332	2641	21.88231605							615.7	66.32373619						
6673.6	5.102691895	2661	21.12626408							620.6	59.28420429						
6720.8	6.909451343	2680.5	16.71816454							625.5	57.66132408						
6772.4	11.19014579	2701.6	19.22210373							630.3	59.07430941						
6815.9	9.337572479	2719.9	24.51002684							634.9	61.83740069						
6868.9	6.502701914	2740.4	25.91694168							639.4	63.29591051						
6914.7	7.013417424	2759.3	24.11012761							644.3	58.19451805						
6961.4	9.000996546	2777.6	27.82754647							649.1	62.05065071						
7007.5	8.654207133	2797.5	26.83279288							654.3	59.74304712						
7055.8	11.00776318	2817.5	28.12030498							659.4	64.24204817						
7104.9	4.211703399	2837.4	21.54997019							671.9	27.53848342						
7159.6	6.792228044	2857.2	21.48112004							683.5	28.26879053						
7211.5	8.561656592	2879	21.21469553							694.8	27.52209034						
7260.1	12.0517952	2898.7	32.03231033							706	24.778509						
7311.5	7.463917886	2918.7	27.14845906							717.2	21.79213857						
7361.3	9.326887697	2939.9	18.42979538							730.1	19.7835474						

Misinterpreting carbon accumulation rates in records from near-surface peat

Dylan M. Young, Andy J. Baird, Dan J. Charman, Chris D. Evans, Angela V. Gallego-Sala, Peter J. Gill, Paul D. M. Hughes, Paul J. Morris, Graeme T. Swindles

Supplementary Table S1. Example carbon accumulation records for tropical, temperate, and Arctic peatlands

(bp = before present, C = carbon accumulation rate)

Tropical						Temperate						Arctic					
Sebangau1_bp	Sebangau1_C	Sebangau3_bp	Sebangau3_C	Sebangau4_bp	Sebangau4_C	Dead_Island_bp	Dead_Island_C	Malham _Tarn_bp	Malham_Tarn_C	Petite_Bog_bp	Petite_bog_C	Marooned_bp	Marooned_C	Toolik_TFS1_bp	Toolik_1_C	Toolik_2_bp	Toolik_2_C
7414	8.737229201	2959.4	27.40359631							744.3	18.72849993						
7463.7	6.414083099	2978.4	40.58724047							758.5	22.54899959						
7513.7	13.24861106	2997.5	36.67936609							771.6	28.6250722						
7562.4	12.48310717	3019	25.84383621							784.1	29.48247774						
7609.9	13.60788707	3040.2	21.90419834							796.7	28.73590688						
7659.1	10.2753144	3060.8	18.34952341							810.1	26.47600813						
7707.8	12.6476542	3081.8	28.59464538							824.8	23.63152808						
7755.5	13.40354347	3101.9	27.83079737							838.1	23.96457613						
7803.3	10.01188676	3121.1	23.68004284							851.7	21.37484477						
7853.8	13.28945431	3141.3	31.14026416							865.1	23.72067431						
7902.4	12.38838557	3161.5	27.92588363							878.7	25.43696567						
7946.9	12.29528967	3182	29.53127001							892.7	21.24209168						
7998.9	9.533169202	3201	32.18505461							905.3	19.83072783						
8041.9	13.06187099	3221.4	24.66448638							917.1	21.46050847						
8092.1	10.65223532	3240.8	31.55014275							930.7	18.85647354						
8140.4	11.19000747	3263.2	20.8566793							945.2	17.11797673						
8194.4	10.83709469	3283.2	29.35879856							959.3	17.01695155						
8240.1	12.17481072	3303	30.75184474							973.1	22.07545265						
8292	10.21635066	3322.8	25.83788909							985.7	29.41169952						
8339.2	11.14712799	3344.3	30.11337104							999.2	26.2862998						
8390.4	9.981139874	3364.7	33.36681549							1013.7	23.38046108						
8437.8	12.0309547	3385.4	32.18024932							1029.3	20.05813065						
8486.1	10.50737553	3403.7	40.05249229							1044.9	18.41185061						
8539.1	11.81767314	3423.7	27.32494604							1059.8	18.32299133						
8592.3	8.912186136	3445.2	29.94719904							1071	23.11930854						
8643.6	11.60764683	3466.8	26.02018008							1082.5	22.19280828						
8692.1	8.851514154	3486.6	34.86441464							1092.9	24.18190551						
8746.5	4.717926681	3507.9	24.36117239							1103.2	26.25116591						
8793.7	6.201948241	3528.5	32.63002402							1113.5	28.09894267						
8844	5.130630507	3548.9	35.13569016							1123.1	27.07214638						
8894	3.621416611	3571.6	47.09839042							1132.7	24.07218142						
8943.4	6.039219283	3591.1	66.53133406							1141.8	25.16115805						
8998.9	6.760995356	3611.1	57.54289068							1151.7	22.91085037						
9046.5	8.100236046	3630.3	58.26359192							1160.2	30.52583034						
9096.7	7.888972164	3650.3	44.70149561							1169.5	31.43116587						
9144.9	8.43570006	3669.1	55.23175579							1178.9	26.60590022						
9197.4	7.948462028	3689	27.04046015							1188.4	21.89618955						
9247.6	8.528026761	3707.5	35.86364197							1198.2	20.91280511						
9299.1	8.525025315	3727.2	39.35935237							1207.7	21.25190033						
9350.6	8.739608493	3746.7	41.83116377							1217.4	20.24520129						
9398.7	13.27792003	3767.5	43.2509234							1227.3	19.19380117						
9448.4	11.34800574	3787	52.73318663							1236.7	28.98585751						
9496.4	11.76673374	3806.9	47.41114038							1245.4	40.82342843						
9549	9.153826827	3826.6	37.28690504							1253.9	36.01417381						
9598.4	11.21701029	3847.3	49.78001567							1263.4	27.27867771						
9646.9	10.5363398	3866.8	37.66009074							1272.5	27.85134642						
9696.8	10.09038331	3887.9	31.92398312							1281.2	28.47723202						
9749.9	8.586510523	3908.2	39.06052793							1289.6	29.33741386						
9799.4	11.03017926	3928.6	33.9772891							1297.8	29.8926057						
9851.7	11.42955375	3947.6	31.0454625							1306.9	28.25674517						
9906.2	8.798266606	3967.8	27.8738535							1316.5	28.04039608						
9956.5	9.237098811	3987.7	26.263667							1326	35.6454101						
10010	8.611920445	4007.8	28.19965751							1334.9	45.99209695						
10063	11.57733942	4028.5	25.20324661							1343.6	40.02400523						
10110.9	10.22187897	4047.4	30.08497473							1353.4	29.29179359						
10158	11.27250197	4067.2	31.14283916							1363.4	27.1898068						
10214	7.972747625	4087.6	25.08000255							1373	26.75495136						
10262.8	11.70056193	4107.8	25.75981008							1383.1	28.44180918						

Misinterpreting carbon accumulation rates in records from near-surface peat

Dylan M. Young, Andy J. Baird, Dan J. Charman, Chris D. Evans, Angela V. Gallego-Sala, Peter J. Gill, Paul D. M. Hughes, Paul J. Morris, Graeme T. Swindles

Supplementary Table S1. Example carbon accumulation records for tropical, temperate, and Arctic peatlands

(bp = before present, C = carbon accumulation rate)

Tropical						Temperate						Arctic					
Sebangau1_bp	Sebangau1_C	Sebangau3_bp	Sebangau3_C	Sebangau4_bp	Sebangau4_C	Dead_Island_bp	Dead_Island_C	Malham _Tarn_bp	Malham_Tarn_C	Petite_Bog_bp	Petite_bog_C	Marooned_bp	Marooned_C	Toolik_TFS1_bp	Toolik_1_C	Toolik_2_bp	Toolik_2_C
10310.9	15.62992637	4126.2	22.9225822							1392.8	32.84425954						
10365.5	9.71225727	4146.8	30.09770358							1401.9	31.50331527						
10417.6	12.34543821	4167.2	28.24874654							1411.2	27.47407173						
10470.2	14.06935398	4189.1	26.98085553							1420.9	27.10186568						
10521	10.31933741	4209.6	29.38230326							1430.4	28.43956516						
10573.3	8.687113388	4228.9	26.91692133							1439.4	30.21608592						
10621.8	13.34117309	4250.2	30.40169566							1448.3	30.75477731						
10671.2	14.18265689	4269.6	34.68367729							1457.8	29.97866802						
10722.3	10.93046303	4287	39.24522395							1467.8	29.55942165						
10765.5	12.93129248	4304.9	35.6283835							1477.7	32.67520854						
10814.9	10.02758051	4322.3	31.44284684							1487	37.91379809						
10864.9	10.0824765	4341.9	24.37185569							1498.3	32.11546785						
10914.1	12.31597013	4362.5	26.15177718							1510.4	30.82322777						
10963.4	10.27801323	4382.2	26.61992465							1522.3	30.04610021						
11015.3	9.953793958	4402.1	30.96930024							1533.1	31.66713726						
11063.7	11.40916785	4422.1	25.59232379							1542.7	31.08802794						
11116.7	10.89462721	4443	25.80826785							1555.9	19.3900008						
11167.1	11.62721634	4463.5	24.13339168							1569.1	18.28020144						
11218.8	10.06818779	4484.4	25.15786885							1581.5	18.29544548						
11267.9	9.470837912	4503.1	28.18061937							1593.5	17.94830172						
11319.8	7.539508115	4522.8	30.68389359							1604.9	17.89769547						
11371.8	7.383427884	4542.7	36.11614899							1619.4	14.76858021						
11419.6	7.128992017	4562.5	29.01390146							1633.9	15.46370468						
11468.9	5.43293577	4582.3	29.57865544														
11523.9	5.79974911	4603.8	23.48524513														
		4624.2	35.12249831														
		4645	34.15978567														
		4664.2	39.15787658														
		4684.7	29.65182667														
		4705.1	32.83579407														
		4725	31.32918141														
		4745.4	31.46801589														
		4765.4	26.53026098														
		4784.1	34.42459125														
		4806.2	31.47366935														
